# Nicotine Inhibits Memory CTL Programming

**DOI:** 10.1371/journal.pone.0068183

**Published:** 2013-07-02

**Authors:** Zhifeng Sun, Kendra Smyth, Karla Garcia, Elliot Mattson, Lei Li, Zhengguo Xiao

**Affiliations:** Department of Animal and Avian Sciences, University of Maryland, College Park, Maryland, United States of America; Uniform Services University of the Health Sciences, United States of America

## Abstract

Nicotine is the main tobacco component responsible for tobacco addiction and is used extensively in smoking and smoking cessation therapies. However, little is known about its effects on the immune system. We confirmed that multiple nicotinic receptors are expressed on mouse and human cytotoxic T lymphocytes (CTLs) and demonstrated that nicotinic receptors on mouse CTLs are regulated during activation. Acute nicotine presence during activation increases primary CTL expansion *in vitro*, but impairs *in vivo* expansion after transfer and subsequent memory CTL differentiation, which reduces protection against subsequent pathogen challenges. Furthermore, nicotine abolishes the regulatory effect of rapamycin on memory CTL programming, which can be attributed to the fact that rapamycin enhances expression of nicotinic receptors. Interestingly, naïve CTLs from chronic nicotine-treated mice have normal memory programming, which is impaired by nicotine during activation *in vitro*. In conclusion, simultaneous exposure to nicotine and antigen during CTL activation negatively affects memory development.

## Introduction

Nicotine is a major alkaloid in tobacco products and is the primary compound responsible for tobacco addiction. Due to consistent integrated efforts worldwide, public awareness of the detrimental effects of cigarette smoking (CS) has been dramatically increased. In an attempt to quit or reduce smoking-related harm, many smokers have tried cigarette cessation therapies or smokeless tobacco products, most of which deliver nicotine but reduce other CS chemicals, such as the carcinogen NNK [1]–[8]. Whether it is being delivered in the form of chewing tobacco, an electronic cigarette, a lozenge, or a dermal patch, nicotine is a major chemical used by millions of people worldwide. Given its status, it is surprising how little is known about nicotine’s influence on the immune system. For example, its effects on the function of cytotoxic T lymphocytes (CTLs), a critical arm of adaptive immunity in fighting cancer and infections, are largely unknown.

To become fully functional, naïve CTLs must be activated during early infection. Activated CTLs clonally expand and acquire effector functions before entering the contraction phase in which 90–95% of them die by apoptosis. The remaining 5–10% become long-lived memory CTLs, which protect the host against re-infection by the same or similar pathogens [9]–[12].

Inflammatory cytokines are induced in early infection [13] and can provide a third signal to CTLs for full activation both *in vitro* and *in vivo* [11], [14]–[21]. The activation of CTLs is impaired and the memory response is abolished in vaccinia virus and *Listeria monocytogenes* infections when IL-12 and Type I IFN receptors are lacking [22]. Together with antigen and costimulation, IL-12 induces CTLs to produce functional molecules such as IFNγ and granzyme B [18], [23]. More importantly, IL-12 can program CTLs to become memory cells [22], [24], and this IL-12-driven memory programming can be upregulated by inhibition of the mammalian target of rapamycin (mTOR) [24], [25], indicating utilization of a major pathway of cell growth regulation [26], [27].

Nicotine shares nicotinic acetylcholine receptors (nAChRs) with the neurotransmitter acetylcholine [1], [3], [28], [29]. Sixteen nAChR subunits are expressed on mammalian neurons and muscle cells, five of which arrange to form heteromeric or homomeric pentamers [29], [30]. The pentamers have an ion channel in their center, which becomes permeable to selective ions such as calcium and sodium upon nicotine binding [6]. In addition to neurons and muscle cells, nAChRs are detected in many other cell types, including bronchial epithelial cells, adipocytes and keratinocytes [31]. nAChRs are also expressed on cells of the immune system, including lymphocytes [32], [33], macrophages and dendritic cells [31]. According to one recent report, both mouse CD4 and CD8 T cells express nAChRs [34]. Qian *et al.* described that CD3 plus CD28 stimulation changes the expression of nAChRs at the transcriptional and translational levels in CD4 and CD8 T cells [34]. Despite this insight, whether nicotine affects CTL memory programming is not known.

In this report, we found that nAChRs are extensively expressed in CTLs of both mice and humans. The nAChRs in mouse CTLs are composed mainly of α2β1β2. Although nicotine did not affect CTL activation, it inhibited IL-12-driven memory CTL programming by reducing memory CTL numbers in all tissues and marginally altering memory phenotype. The presence of nicotine ablated rapamycin’s positive effects on memory programming by IL-12. In contrast, chronic nicotine treatment of donor mice did not affect the ability of naïve CTLs to respond to IL-12-driven memory programming, as long as nicotine was absent during the initial T cell activation period. Therefore, nicotine negatively regulates memory CTL programming during activation, suggesting that use of nicotine early after vaccination may be harmful to vaccine efficacy.

## Results

### Multiple nAChR Subunits are Expressed in CTLs from Humans and Mice

**Figure 1:**
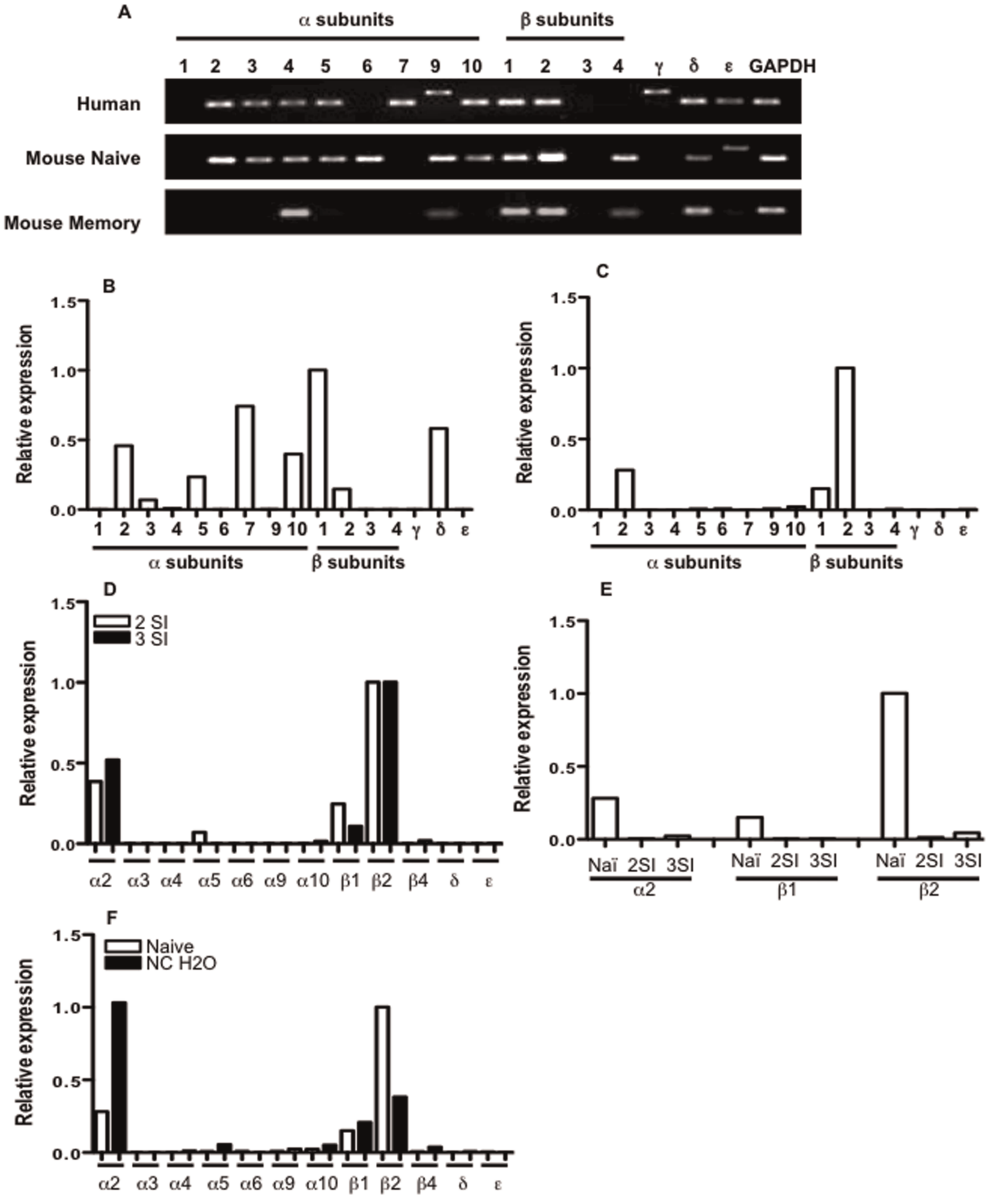
Expression of nAChRs in CTLs from human and mice. Purified RNA from human and mouse CD8 T cells were examined for nAChR subunits using PCR. A. PCR results (40 cycles, all PCR products were confirmed by sequencing). Memory OT-I cells were obtained as described in the text and as previously reported [35], [83]. B–C. Quantitative PCR was performed on RNA from human CD8 T cells (B) or murine naive CD8 T cells (C). Expression of all subunits was relative to β2 (the highest expression) in murine naïve CTLs (C), which was designated as 1. D. Purified naïve OT-I cells were stimulated with 2SI or 3SI (2SI plus IL-12) for 3 days, and expression of nAChRs was compared relatively to β2 in each treatment (2SI and 3SI). E. The expression of 3 dominant nAChR subunits in 2SI and 3SI treatments was compared to naïve OT-I using the housekeeping gene GAPDH as internal control. Subunits α1, α7 and β3 were not detectable in D–E. F. CD8 OT-I cells were purified from naïve OT-I mice or OT-I mice treated with nicotine water at 200 µg/ml for 60 days to quantify the expression of nAChR subunits. Data are representatives of at least three experiments with similar results.

To examine the profile of nicotinic receptors in human CD8 T cells, regular and quantitative PCRs were performed on purified RNA from CD8 T cells of healthy human adults. Of 16 known human nAChR subunits, 13 were expressed and confirmed by regular PCR and sequencing (Fig. 1A and data not shown). Among them, α2, α5, α7, α10, β1, β2 and δ are expressed at a relatively high levels (Fig. 1B). The presence of α7 and β1, with an absence of α1 and α4, is different from typical neuronal and muscle nAChRs in humans [3], [5], [29]. For mouse CTLs, naïve CD8 T cells were purified from OT-I mice [25], [35], and 12 nAChR subunits were detected (Fig. 1A). Mouse CTLs differed from human CTLs in the lack of α7 and γ, and expression of α6 and β4. There were 3 highly expressed nAChRs in naïve mouse CD8 OT-I cells: α2, β1 and β2, with β2 about 4–6 times higher than α2 and β1 (Fig. 1C). This suggests that the nAChRs in naïve mouse CTLs may be mainly comprised of α2, β2, and β1 subunits. These data are consistent with a recent report that α2 is the dominant α subunit [34].

We asked if mouse memory CTLs express the same pattern of nAChRs as naïve CTLs. B6 mice received naïve OT-I cells and were infected with LM-OVA the next day. Sixty days after infection, spleen samples were harvested, and memory OT-I cells were purified by positive selection as previously reported [35]. Surprisingly, these memory CTLs only expressed 6 of the 12 nAChRs that were present on naïve CTLs. Although not quantitative, the absence of α2 and presence of α4 suggests that previous infection changes nAChR composition. This could be related to the interaction of the CTL with certain inflammatory cytokines, such as IL-12, that are produced during infection. In summary, we confirmed that nAChRs are extensively expressed on both mouse and human CTLs.

### nAChR Expression is Regulated by CTL Activation and Chronic Nicotine Treatment

We then asked if CTL activation and chronic nicotine exposure could affect nAChR expression. Purified OT-I cells were cultured and stimulated with 2 signals (2SI: antigen and B7) or 3 signals (3SI: 2SI plus IL-12) for 3 days [22], [25]. The profile of nAChR subunits was similar in 2SI and 3SI stimulated cells, with α2, β1 and β2 as the dominant subunits (Fig. 1D). This is further evidence that α2, β2 and β1 may form unique nAChRs on mouse CTLs and that they are possibly regulated as a group. However, following activation, the expression levels of these three dominant subunits were reduced to 5% or less of their relative expression levels on naïve OT-I (Fig. 1E). We did not see upregulation of any subunits as reported by Qian et al [34], which may be due to the length of culture (their 5-day vs our 3-day culture) or the method of stimulation (they used anti-CD3 stimulation). In addition, when OT-I mice were treated with nicotine in drinking water (200 µg/ml) for 2 months [36], [37], the three dominant α2, β2 and β1 subunits continued to show the highest relative expression. However, α2 expression increased about 4-fold compared to control mice given normal drinking water, whereas β2 dropped to about half of the levels seen in control mice (Fig. 1F). These data suggest that nAChRs in CTLs can be regulated by antigen stimulation and chronic nicotine treatment, which may be related to their responsiveness to nicotine.

### Nicotine Inhibits CTL Memory Programming but not Activation

**Figure 2:**
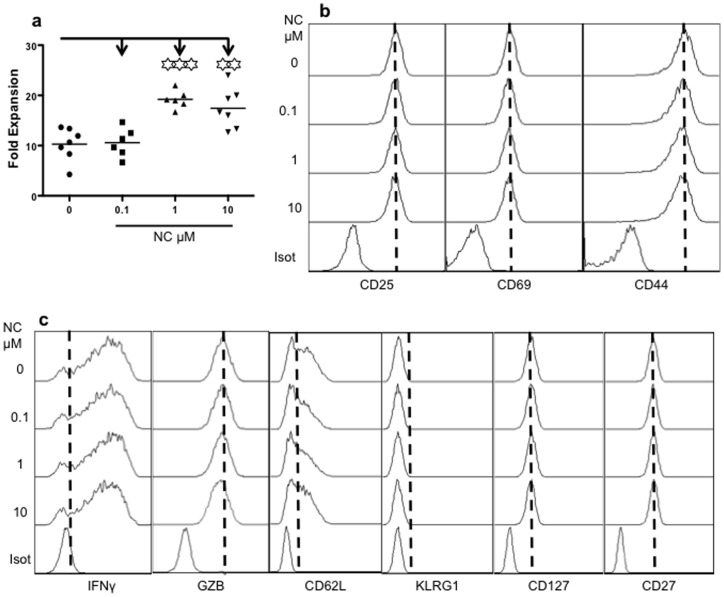
Nicotine does not affect CTL activation. Purified OT-I cells were cultured for 3 days with 3SI in the presence of nicotine at different concentrations. A. Comparison of fold expansion was calculated as the cell yield at day 3 divided by initial input cells at day 0. B. Cells were harvested to examine activation markers. C. Comparison of IFNγ, granzyme B (GZB) and other surface molecules. Data are representatives of at least five experiments with similar results. Asterisks indicate statistical significance. *, P<0.05; **, P<0.01; ***, P<0.001.

The presence of multiple nAChR subunits on naïve mouse CTLs suggests that nicotine may affect CTL function by binding to its receptors. We and others have found that IL-12 can drive full activation of CTLs and program memory CTLs *in vitro* [11], [18], [22]. We sought to understand if this function of IL-12 could be affected by nicotine. Briefly, sorted naïve OT-I cells were stimulated with 3SI for 3 days [22]. Nicotine was added simultaneously at 3 different concentrations covering a 100-fold range from 0.1 to 10 µM to mimic different levels of nicotine use[34], [37]–[39]. Activated OT-I cells were harvested at day 3 for analysis. There was a significant increase in CTL expansion at nicotine concentrations above 1 µM, but no difference between 1 and 10 µM (Fig. 2A). However, no change was observed in the expression of activation markers (CD25, CD69 and CD44) at any nicotine concentration (Fig. 2B). The production of IFNγ was not affected by nicotine, but we consistently noticed a marginal reduction in the production of granzyme B at 10 µM of nicotine (Fig. 2C). There was no difference in the expression of CD62L, KLRG1, CD127 and CD27 at different nicotine concentrations (Fig. 2C).

**Figure 3:**
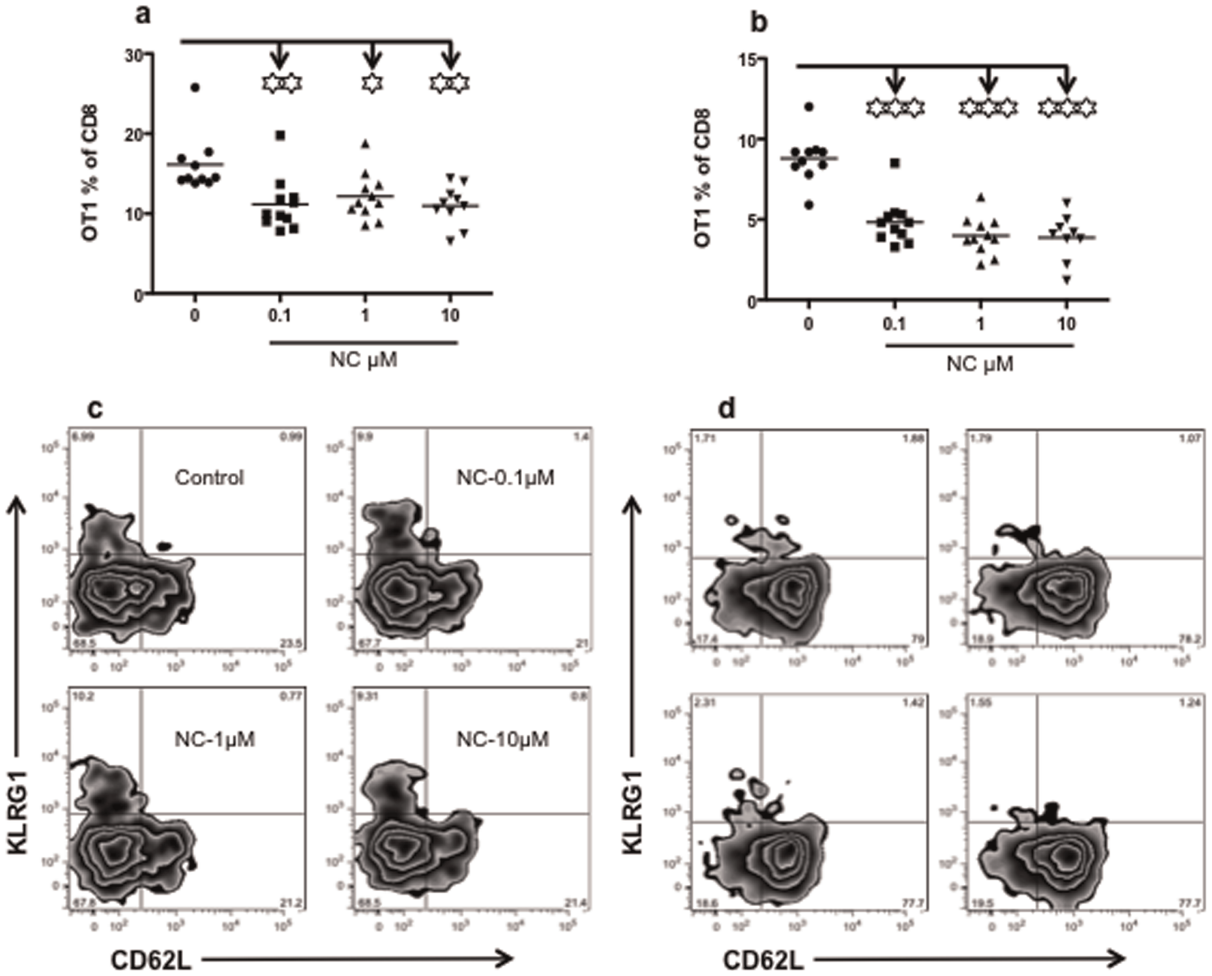
Nicotine reduces CTL expansion after transfer into recipients. Purified OT-I cells were cultured for 3 days with 3SI in the presence of nicotine at different concentrations. Three days after stimulation, CTLs were harvested and transferred into B6 recipients at 10^6^ cells/mouse. A–B. Comparison of percentage of OT-I cells in total CD8 cells in blood at day 5 (A) and 14 (B) post transfer. C–D. Comparison of the phenotype of OT1 cells in blood from mice at day 5 (C) and 14 (D) post transfer. Data are obtained from one experiment with 10 to 11 mice per group. Asterisks indicate statistical significance. *, P<0.05; **, P<0.01; ***, P<0.001.

**Figure 4:**
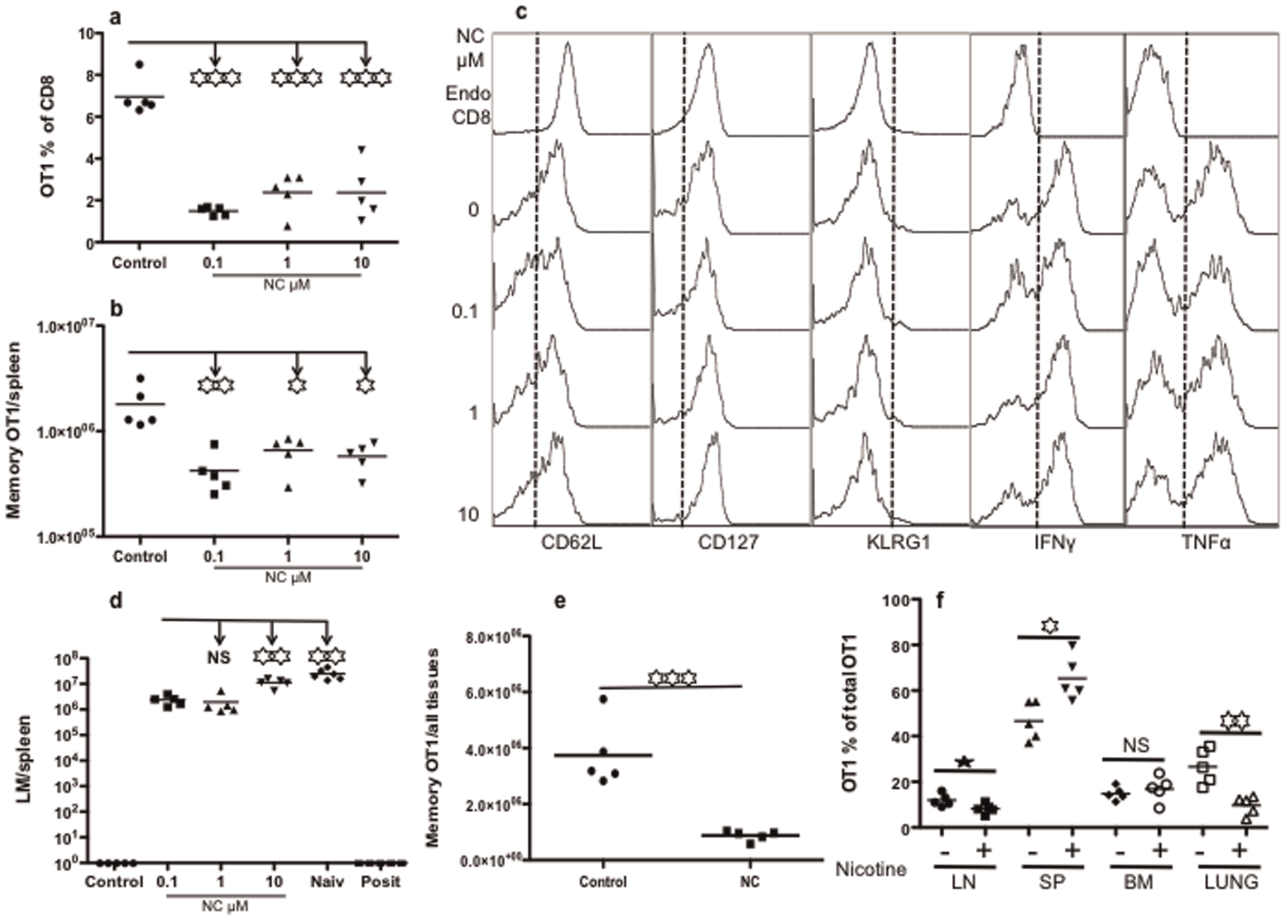
Nicotine impairs memory CTL programming. A–D: Purified OT-I cells were cultured for 3 days with 3SI in the presence of nicotine at different concentrations. Three days after stimulation, CTLs were harvested and transferred into B6 recipients at a concentration of 10^6^ cells/mouse. At day 30 post-transfer, memory CTLs were examined in the spleen. A. Comparison of percentage of OT-I cells in total CD8 cells in spleens. B. Comparison of number of memory CTLs in spleen. C. Comparison of phenotype of memory CTLs in spleen. The dashed line indicates gating for positive population, and endogenous CD8 T cell in recipients are shown as control. D. Recipient mice were challenged with LM-OVA as previously reported [25] and protection was compared to 0.1 µg/ml nicotine treatment at 3 days after challenge in spleen. “Positive” control mice were VV-OVA infected memory B6 mice. B6 mice were first transferred with 10^5^/mouse naïve OT1 cells, which were infected with VV-OVA the next day, then sat for 30 days. These VV-OVA infected memory mice have effective memory CTLs against LM-OVA rechallenge, as we reported previously [22]. E–F: *In vitro* stimulated cells with 3SI in the presence or absence of nicotine at 10 µM were transferred into B6 mice for 30 days, and total number of memory OT-I was examined in peripheral lymph nodes, spleen, bone marrow and lung. E. Comparison of total memory CTLs from different tissues. F. Comparison of memory CTL distribution in tissues. The percentage was calculated by dividing the number of memory OT-I in each individual tissue by the total memory OT-I from examined tissues in each mouse. The experiment was repeated three times and similar results were obtained. Asterisks indicate statistical significance. *, P<0.05; **, P<0.01; ***, P<0.001.

To determine if acute nicotine exposure had any effects on IL-12 memory programming, 10^6^ harvested CTLs were transferred into naïve B6 mice. Because there is no stimulation provided to recipients, differences in the final outcomes should be only due to differences of *in vitro* stimulation. 3SI activated CTLs went through drastic expansion 5 days after transfer (Fig. 3A), consistent with our previous report [25]. However, nicotine treatment led to reduced expansion (about 30%) (Fig. 3A), and this population declined faster than controls during contraction phase (day 14 after transfer, Fig. 3B). There was no much difference in the expression of CD62L and KLRG1 (Fig. 3C and D). Memory OT-I cells were examined in several major tissues 30 days after transfer. Nicotine treatment during initial T cell activation significantly reduced memory OT-I cells about 4-fold (Fig. 4A and B), suggesting that acute nicotine treatment impairs memory programming. There were no significant differences in the production of IFNγ and TNFα in memory CTLs between groups (Fig. 4C). Consistently, nicotine pretreatment led to significantly reduced protection to LM-OVA challenge compared to controls (Fig. 4D). Although protection is positively associated with the number of memory CTLs, the 3–4 fold difference in memory T cell numbers between control 3SI and nicotine-treated CTLs could not solely explain the drastic reduction (6–7 logs) in protection (Fig. 4A–B and 4D). Furthermore, even though the number of CTLs was similar among different nicotine concentrations, there was a significant increase in LM growth in mice receiving cells treated with 10 µM versus 0.1 µM of nicotine. This indicates that increased nicotine concentrations impair the protective ability of memory CTLs (Fig. 4D).

The reduced number of memory CTLs in the spleen was not due to biased migration to other tissues. The total number of memory CTLs from peripheral lymph nodes, spleen, bone marrow and lung was about 4 fold higher in the controls versus the nicotine-pretreated group (Fig. 4E). However, of the total OT-1 T cells recovered, the highest percentages of nicotine-pretreated CTLs were found in the spleen, and these values were elevated when compared to splenic OT-1 percentages in control mice (Fig. 4F). Notably, the relative percentages of nicotine-pretreated OT-1 T cells in the lung were significantly decreased relative to control mice (Fig. 4F), which may be related to the susceptibility of tobacco users to respiratory infections and lung cancer [40], [41]. It is worth noting that nicotine pretreatment significantly upregulated CD127 on memory CTLs in the blood (Fig. S1A), lymphoid and nonlymphoid tissues (Fig.S1B). Memory CTLs in other tissues manifested differences in the expression of CD62L, production of IFNγ and TNFα (Fig. S1C–D), which is consistent with CTL heterogeneity in different tissues as we reported previously [42]. These data indicate that acute nicotine treatment during CTL activation inhibits the CTL expansion capacity and CTL memory differentiation.

### Nicotine does not Affect T-bet Expression

**Figure 5:**
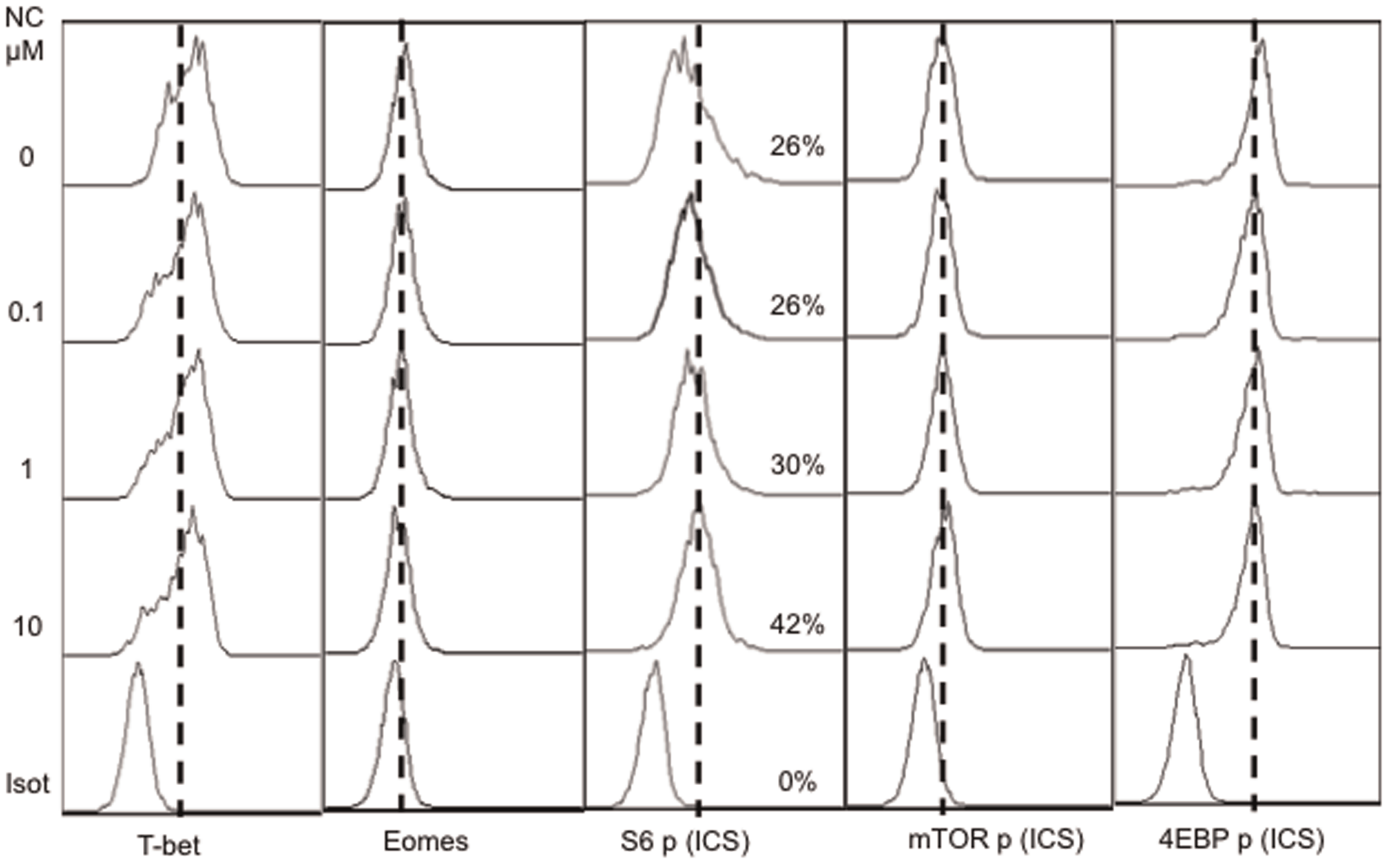
Nicotine effects on T-bet expression and mTOR signaling. Sorted OT-I cells were stimulated with 3SI (antigen+B7+IL-12) in the presence of nicotine at different concentrations. Programmed CTLs were harvested at 3 days post-stimulation and analyzed for expression of T-bet, Eomes, S6, mTOR nad 4EBP. These are representatives of three independent experiments with similar results.

T-bet is a critical transcription factor responsible for the differentiation of Th1 cells and the production of IFNγ [43]–[45]. The balance between T-bet and the transcriptional activator Eomes has important implications for memory differentiation [24], [46]. To examine if the suppressive effects of nicotine on memory CTL programming were related to alterations in T-bet and Eomes expression, sorted naïve OT-I cells were stimulated under various conditions for 3 days and subsequently analyzed. There was no difference in T-bet expression among any of the nicotine concentrations and only a marginal decrease in Eomes expression at the highest nicotine concentration (Fig. 5). It has recently been reported that the mTOR pathway is activated in IL-12 stimulation of CTLs [24]. The presence of nicotine did not alter the phosphorylation of mTOR, but phosphorylation of 4EBP, another component of the mTOR pathway, was slightly decreased (Fig. 5). In contrast, phosphorylation of S6, a protein responsible for synthesis during cell growth, was upregulated with increasing nicotine concentrations (Fig. 5). This may indicate that the presence of nicotine enhances protein synthesis during CTL activation, which is consistent with the increased expansion seen in Fig. 1A. Thus, nicotine may affect CTL expansion through cell growth pathways other than canonical mTOR signaling or regulation of T-bet or Eomes, such as by PI3K pathway through PDK1 [47], [48] or Akt [49].

### Nicotine Abolishes the Regulatory Function of Rapamycin on Memory Programming

**Figure 6:**
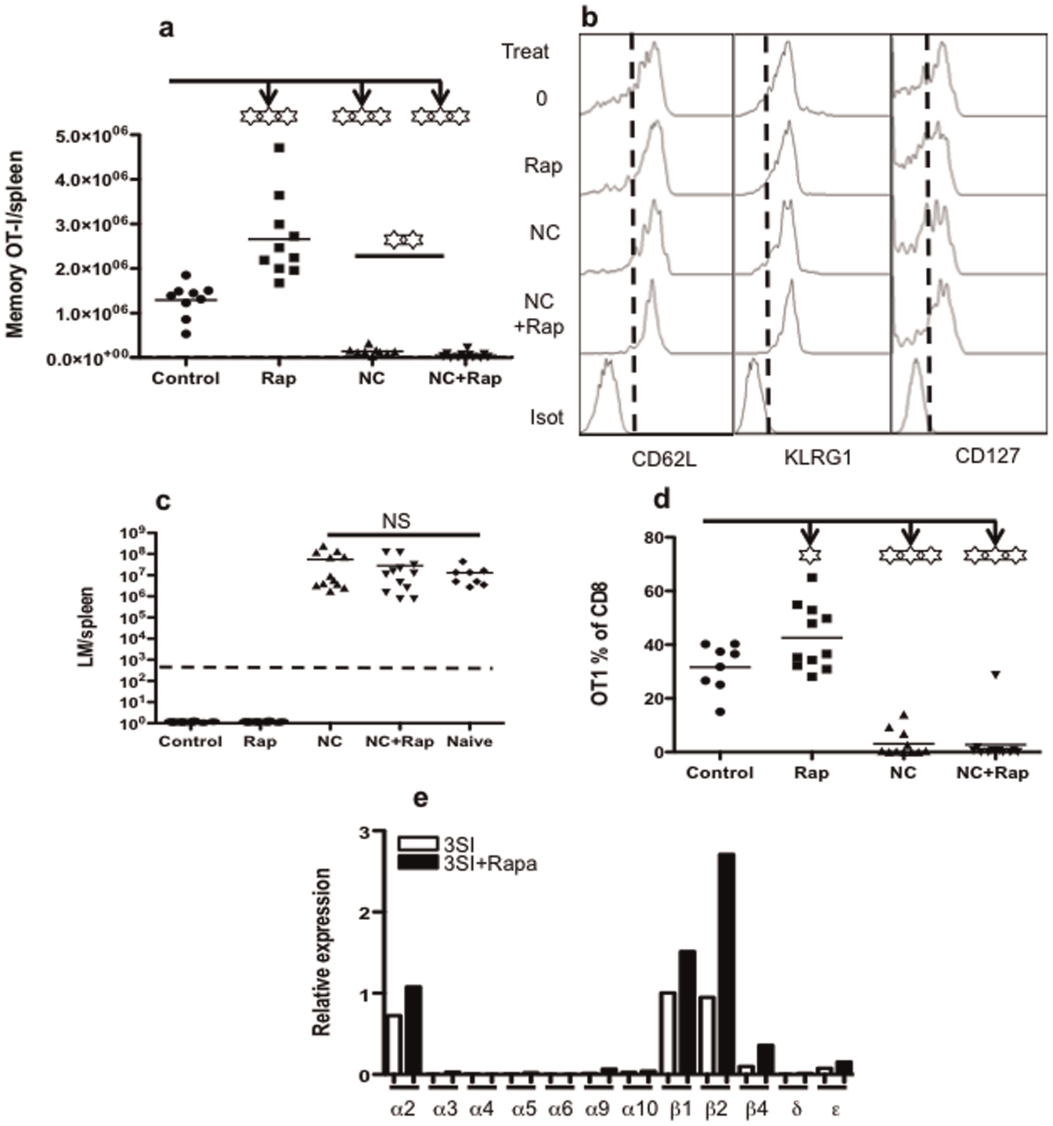
Nicotine abolishes the regulatory function of rapamycin on memory programming. Sorted OT-I cells were stimulated for 3 days with 3SI in the presence or absence of nicotine at 10 µM. Rapamycin was used in combination with or without nicotine. Cells were harvested and transferred into recipient B6 mice at a concentration of 10^6^ cells/mouse. Memory CTLs were examined 40 days post-transfer. A. Comparison of memory CTLs in spleen. B. Comparison of the phenotype of memory CTLs in spleen. C. Comparison of memory protection against LM-OVA challenge in different memory mice in Panel A. D. Secondary expansion of memory CTLs 3 days post-LM-OVA challenge. These animals were the same as mentioned in Panel C. The results are representatives of two separate experiments with similar results. Asterisks indicate statistical significance. *, P<0.05; **, P<0.01; ***, P<0.001. E. Sorted OT-I cells were stimulated for 3 days with 3SI in the presence or absence of rapamycin, and expression of nAChRs was compared relatively to β1 in 3SI stimulation using quantitative PCR. Dotted lines in A and C indicate the detection limits. The results are representatives of two separate experiments with similar results.

Because nicotine increased S6 phosphorylation, and S6 is a downstream target of mTOR pathway [50]–[52], we hypothesized that the inhibition of mTOR by rapamycin would suppress S6 activity and thus reverse the negative effects of nicotine on memory programming. To test this, naïve OT-I cells were stimulated in the presence of nicotine, rapamycin, or both. Cells were harvested after 3 days of stimulation and transferred into B6 recipients. Forty days after transfer, rapamycin pretreatment significantly enhanced memory CTLs in the blood (Fig. 6A), which is consistent with previous reports [24], [25]. Similar to results in Fig. 4A and B, nicotine pretreatment dramatically reduced memory CTL number (Fig. 6A). However, the presence of rapamycin did not reverse the negative effects of nicotine, but significantly exacerbated them (p<0.01) (Fig. 6A). This suggests that rapamycin may change the responsiveness of CTLs to nicotine.

To test their protective ability, the mice transferred with programmed OT-I cells for 40 days were challenged with LM-OVA [25]. As observed before (Fig. 4D), the mice receiving IL-12-conditioned OT-1 cells with or without rapamycin were fully protected from LM-OVA challenge. When nicotine was present during stimulation, rapamycin failed to rescue impaired memory programming (Fig. 6C). In the LM-OVA challenged mice, CTLs showed significantly higher expansion in the rapamycin pretreated group compared to controls, but expanded poorly in both the nicotine and nicotine plus rapamycin groups (Fig. 6D). This is consistent with the protection data shown in Figure 6C. Therefore, rapamycin treatment is not sufficient to rescue CTLs from nicotine-associated defects. To test if the presence of rapamycin could affect the expression of nicotinic receptor expression, sorted OT-I cells were stimulated with 3SI in the presence or absence of rapamycin for three days, which were then harvested for quantitative PCR analysis. To our surprise, rapamycin enhanced the expression of α2, β2, and β1 subunits (β4 and ε also increased) (Fig. 6E). This suggests that the failure of rescue function from rapamycin may be related to the increased responsiveness of CTLs to nicotine due to augmented nicotinic receptor expression by rapamycin.

### Chronic Exposure to Nicotine does not Alter the Ability of Memory Programming in Naïve CTLs

**Figure 7:**
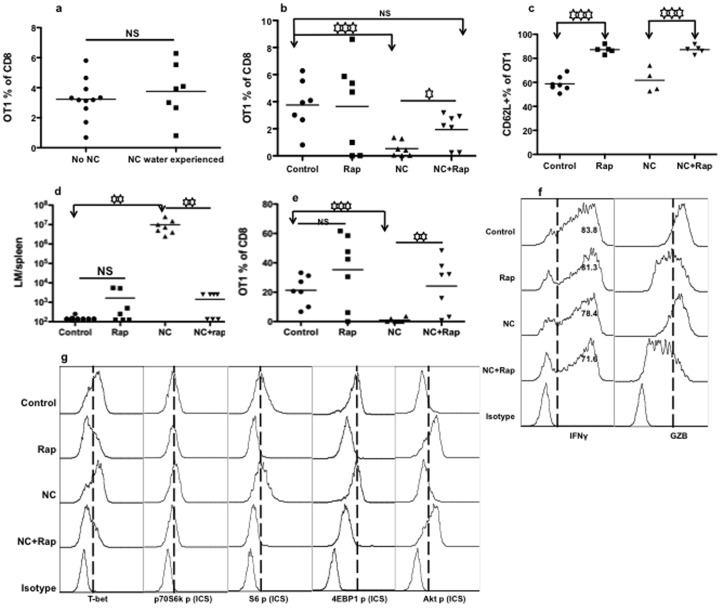
Chronic exposure to nicotine does not change naïve CTL’s memory programming. OT-I transgenic mice were given drinking water supplemented with nicotine at 200 µg/ml for 60 days [34], [37]–[39]. Naïve OT-I cells were purified from the nicotine treated OT-I mice or un-treated control mice and stimulated for 3 days with 3SI in the presence or absence of nicotine at 10 µM. Rapamycin was used with or without nicotine. Cells were then harvested for adoptive transfer at a concentration of 10^6^ cells/mouse for memory differentiation in recipient B6 mice [22], [24], [25] (A–E) or direct examination (F–G). A–C. Analysis of memory CTLs 30 days after transfer. A. Comparison of memory CTLs in blood programmed *in vitro* by IL-12 from control or nicotine experienced OT-I mice. B. Comparison of memory CTLs in blood programmed under different treatments. All of the naïve OT-I CTLs were from nicotine treated donor mice. Data for “NC water experienced” in (A) are the same as “Control” in (B). C. CD62L expression on memory CTLs from blood in (B). D. Memory mice from (B) were challenged with LM-OVA, and protection was compared 3 days after challenge in spleen. E. Memory CTL secondary expansion in the blood 3 days after LM-OVA challenged (the same mice in D) mice. F–G. *In vitro* stimulated cells (for 3 days) were harvested to examine the production of functional molecules (F) or transcription factors and mTOR signaling molecules (G). Data from one experiment is shown, representative of two other experiments with similar results. Asterisks indicate statistical significance. *, P<0.05; **, P<0.01; ***, P<0.001, NS, not significant.

In most scenarios nicotine exposure is chronic. To understand the effects of chronic nicotine experience on the ability of naïve CTLs to differentiate into memory cells, OT-I CTLs from chronic nicotine-treated (administered at 200 µg/ml in drinking water) and non-treated mice were stimulated with 3 signals for 3 days in the presence or absence of nicotine, and were then transferred into recipient B6 mice. Naïve CTLs with or without chronic nicotine exposure were programmed to similar levels of memory CTLs (Fig. 7A) with a mostly central memory phenotype (data not shown), suggesting a normal ability of memory programming in naïve CTLs from chronically exposed mice. Similar to normal naïve OT-Is, nicotine-experienced naïve OT-Is were sensitive to negative regulation by nicotine during T cell activation, resulting in significantly fewer memory CTLs (Fig. 7B). However, unlike our results in naïve OT-I cells (Fig. 6A), impaired memory CTL programming was partially rescued by rapamycin (Fig. 7B). Likewise, rapamycin increased CD62L expression, whereas nicotine did not (Fig. 7C).

The upregulation of memory CTLs by rapamycin was further confirmed by LM-OVA challenge and memory expansion (Fig. 7D–E). After LM challenge, LM growth in the spleen was significantly reduced in the rapamycin pretreated group by 4 logs (Fig. 7D) and memory expansion was significantly higher in the rapamycin plus nicotine group compared to nicotine only (Fig. 7E). It is noteworthy that these chronic nicotine-experienced naïve CTLs responded differently to rapamycin from normal naïve CTLs (Fig. 7B–C and Fig. 6A and 6C).

To investigate if these outcomes were related to the production of effector molecules downstream of T-bet and mTOR signaling pathways, the nicotine-experienced naïve CTLs were examined after stimulation *in vitro* under the same conditions as described in Fig. 7A–E. Nicotine marginally affected the production of IFNγ and GZB (Fig. 7F). Rapamycin reduced IFNγ and GZB production, and the presence of nicotine significantly aggravated this reduction (Fig. 7F and data not shown). There were no significant changes in the expression of activation markers CD25 and CD44 (data not shown). Rapamycin downregulated T-bet to similar levels in the presence or absence of nicotine, and comparable results were obtained for mTOR signaling molecules (Fig. 7G). Therefore, chronic-nicotine-experienced naïve CTLs possess a similar ability to be programmed into memory cells relative to un-exposed controls and rapamycin can partially rescue the inhibitory effects of nicotine.

## Discussion

Nicotine, the main player in perpetuating tobacco addiction, is also used extensively in harm-reduced smoking products and smoking cessation therapies [7], [8], [28], [53]. Nicotine’s addictive nature has made it notorious, yet its benefits include temporary improvement in cognitive functions and anti-inflammatory activities [6], [28], [54]–[57]. Despite widespread usage, little is known about nicotine’s effects on the immune system. CTLs are critical in the control of malignant cells and intracellular pathogens [9], [58], [59]. The mechanisms by which this powerful chemical alters our bodies’ ability to fight intracellular infections and cancers through its influence on CTLs has yet to be carefully evaluated.

Here, we present evidence that nicotine directly affects the functions of CTLs through its receptors. nAchRs are expressed in both naïve and memory mouse CTLs, and their expression is quickly downregulated by CTL activation. Nicotine exposure during the critical time of CTL activation impaired memory programming, ultimately decreasing memory CTL numbers and their ability to protect against pathogen challenge. However, we did not observe a decrease in overall activation (Fig. 2C) nor did we see any significant changes in phenotype or production of functional molecules in memory CTLs (Fig. 4C). In fact, nicotine-conditioned memory CTLs presented a slightly more central memory phenotype in terms of CD62L and CD127 expression (Fig. 4C and data not shown). Nicotine treatment led to reduced expansion of CTLs after transfer (Fig.3A), which should contribute at least partially to the reduced memory formation. Furthermore, the protection ability of the nicotine-experienced memory CTLs was severely suppressed (by about 6 logs) (Fig. 4D), which is not proportional to the difference in numbers of memory CTLs (about 4 fold difference). Therefore, nicotine inhibits memory CTL programming quantitatively and qualitatively. Interestingly, smokers who developed chronic bronchitis and chronic airflow limitation had increased numbers of CD8 in the peripheral airways compared to smokers with out symptoms [60]. Smokers with chronic obstructive pulmonary disease (COPD) had higher number of CD8 T cells in both lung parenchyma and pulmonary arteries compared with asymptomatic smokers and nonsmokers [61].This may be related to the enhanced expansion of CTLs by nicotine (Fig. 2A) and/or to biased migration [62]. However, the increased number of CTLs should not be due to nicotine-induced memory CTLs, because nicotine clearly suppresses memory programming.

Rapamycin is known to enhance memory CTL programming by IL-12 [24], [25]. The presence of nicotine completely abolished this regulatory function of rapamycin (Fig. 6). In fact, rapamycin significantly reduced the memory CTLs even further than nicotine alone (Fig. 6A), which could be related to the direct enhancement of nicotinic receptor expression by rapamycin (Fig. 6E). This suggests that using rapamycin to enhance memory CTLs in vaccination may not work in nicotine users, instead harming the nicotine-influenced memory CTLs. It is a different story for nicotine experienced naïve CTLs. Naïve CTLs from chronic nicotine treated donors were able to generate normal memory CTLs after IL-12 stimulation *in vitro*, and later nicotine *in vitro* treatment similarly suppressed this memory programming (Fig.7). Surprisingly, the presence of rapamycin during activation did not enhance memory programming in 3SI stimulation, but did partially rescue memory programming impaired by nicotine (Fig. 7B, D–E). This could be related to their differences on nicotinic receptor expression regulated by rapamycin (Fig. 6E). The differences of rapamycin regulatory effects on normal versus nicotine treated naïve CTLs does not appear to be related to the rapamycin effects on activation and mTOR signaling. Rapamycin modulated these molecules similarly in both settings regardless of the presence or absence of nicotine (Fig. 7F–G and data not shown). These data clearly demonstrate that nicotine directly inhibits memory CTL programming and imply that using nicotine right after vaccination inhibit the induction of memory CTLs, thus negatively affecting vaccine efficacy.

Although we have established clear evidence that nicotine can directly inhibit CTL memory programming, elucidation of underlying molecular mechanisms is needed and is currently underway. We investigated several traditional pathways involved in memory CTL differentiation, including T-bet/Eomes and mTOR signaling. Upregulated T-bet expression is related to effector CTL function whereas Eomes upregulation drives CTLs toward memory functions [24], [46]. In this study, nicotine did not alter the T-bet/Eomes ratio (Fig. 5). Second, nicotine did not influence mTOR signaling molecules except for S6 (slightly upregulated, Fig. 5). Third, rapamycin exerted different regulatory functions on memory programming in the presence and absence of nicotine, suggesting that nicotine does affect mTOR signaling, but this was not through canonical signaling molecules such as phosphorylation of mTOR. Although gene expression can be regulated at many different stages, regulation by transcription factors is one of the most efficient mechanisms in controlling gene expression. Many transcription factors are involved in CTL memory differentiation, such as Gfi-1, T-bet, eomes, Id2, Blimp-1, XBP-1, Bcl6 and Bcl6b, Mbd2 and Bmi-1 [43], [63]–[70]. Recently, interleukin-10-21-STAT3 was shown to be critical for memory CTL precursor maturation [71] and FOXO1 is required for the T-bet to Eomes switch for memory programming [72]. Interestingly, deficiency of FOXO3 increases memory CTL development by suppressing apoptosis of effectors [73]. With a long list of potential transcription factors, we are currently investigating the change of transcription factors using high throughput approaches such as proteomics. We hope to identify critical candidates, which could be targeted to reverse the detrimental effects of nicotine.

Muscle nAChRs include α1β1δε and α1β1δγ, whereas the most abundant brain nAChRs consist of α7, α4β2 and α3β4 [29]. In this study, we found that α2β1β2 is the dominant receptor type on mouse CTLs and is expressed at the highest level in naive CTLs but reduced to about 5% or less after activation (Fig. 1E). The nAChRs on memory CTLs seem to be different from those in naïve CTLs, in which α4 replaced α2, but β1β2 remained. This change of the α subunit may indicate different binding abilities, suggesting that memory and naïve CTLs may respond differently to nicotine, which warrants future study. The timing and transition of different nAChR subunits from naïve to memory after activation will be investigated in the future. In addition, inflammatory cytokines TNFα and IL-1β can enhance nAChR expression [31], [74]. Nicotine can upregulate nAChRs in neural cells through post-transcriptional mechanisms by affecting cell surface turnover, receptor trafficking and degradation [5], [75]. Although we did not observe any transcriptional upregulation of subunits after stimulation (antigen, cytokine and nicotine, data not shown), our next step will be analysis at the protein level.

It is well known that the vagus nerve can modulate innate immunity by releasing acetylcholine, which binds α7 subunit [55] on macrophages, thus affecting production of inflammatory cytokines such as TNFα and IL-1β [76]. Our data suggest that the vagus nerve may modulate immune effectors directly. Numerous neurotransmitter receptors are expressed by T cells, such as dopamine, glutamate, serotonin, and GnRH-I, GnRH-II, Substance P, and Somatostatin, which affect T cell proliferation, production of cytokines and migration [77], [78]. Surprisingly, the neurotransmitter acetylcholine and its receptors are both detected in human T cells [79], suggesting that T cells are not only able to respond to neurotransmitters, but also the producers of some neurotransmitters [79]. Thus, interactions between T cells and the neural system may be more common than currently thought.

nAChRs are highly permeable to calcium and the calcium signal is strong in nAChR-mediated effects on neurons [56]. In T cell activation, ligation of antigen with the T cell receptor induces production of IP3, which binds to the IP3 receptor calcium channel on the membrane of the endoplasmic reticulum, releasing intracellular calcium stores and causing a transient calcium rise [80]. This depletion of stored calcium leads to the opening of calcium channels on the cell membrane, resulting in a sustained, low amplitude calcium influx, which is essential for NFAT activation and IL-2 transcription [80], [81]. The high expression of nAChRs on naïve CTLs and the binding of nicotine to nAChRs may lead to a rapid calcium influx, which may be harmful to memory CTL programming. More research needs to be done on this aspect.

In summary, our data indicate that simultaneous exposure to nicotine and antigen in the critical window of CTL activation, such as early after vaccination, negatively affects the development of memory CTLs. This can be avoided, even in chronic nicotine users, by temporarily abstaining from nicotine right after vaccination.

## Materials and Methods

### Mice and Reagents

OT-I mice (a gift from Dr. Mescher, University of Minnesota) having a transgenic TCR specific for H-2K^b^ and OVA_257–264_ [82] were crossed with Thy1-congenic B6.PL-Thy1a/Cy (Thy1.1) mice (Jackson ImmunoResearch Laboratories, Bar Harbor ME) and bred to homozygosity. The development of CD8 T cells in all strains appeared normal with respect to numbers, distribution and phenotype (data not shown). Mice were maintained under specific pathogen-free conditions at the University of Maryland, and these studies have been reviewed and approved by the Institutional Animal Care and Use Committee. C57BL/6 mice were purchased from the National Cancer Institute. All directly conjugated fluorescent antibodies were purchased from BD Biosciences, eBioscience or Biolegend. Rapamycin was purchased from EMD (Gibbstown, NJ), and nicotine from Sigma (St. Louis, MO). Purified RNA from CD8 T cells of healthy human adults was purchased from Miltenyi Biotec (Cambridge, MA). Nicotine stock was made in ethanol, and rapamycin stock was made in DMSO. Chronic nicotine treatment was performed by adding nicotine into drinking water at a concentration of 200 µg/ml for two months. This dose of nicotine is based on the range of daily nicotine intake in intermediate and heavy smokers [36], [37].

### Bacteria

Recombinant *Listeria monocytogenes* (a gift from Dr. Jameson, University of Minnesota) expressing full-length secreted ovalbumin (LM-OVA) was used for inoculation of 5×10^5^ CFU/mouse via the i.v. route. Mouse spleens were harvested 3 days after LM-OVA challenge, and LM-OVA was cultured using TSB plates for comparison of protection as in our previous reports [22], [25].

### Naive T cell Purification

Naïve T cell purification was performed as previously reported [22], [25]. Briefly, inguinal, axillary, brachial, cervical, and mesenteric lymph nodes (LNs) were harvested from WT OT-I mice, pooled, and disrupted to obtain a single cell suspension. Cells were incubated with FITC-labeled antibodies specific for CD4, B220, I-A^b^, and CD44. Anti-FITC magnetic MicroBeads (Miltenyi Biotech) were then added and the suspension passed through separation columns attached to a MACS magnet. Cells that did not bind were collected with a purity >95% CD8^+^ cells and <0.5% CD44^hi^ Cells. Purified naive OT-I cells were then sorted to reach a purity close to 100%.

### Real-time RT-PCR

RNA was isolated (Qiagen RNeasy mini kit) and used to synthesize cDNA (Qiagen, QuantiTech Reverse Transcription kit). Quantification was performed on a *MyiQ*™ *Single*-Color Real-Time PCR Detection System (Bio-Rad). Primers used were as follows: Primer sequences of mouse and human nAChRs and GAPDH are listed in Table S1. Details of the real-time PCR conditions used are available upon request.

### Adoptive Transfer and Flow Cytometric Analysis

*In vitro* activated OT-I cells were adoptively transferred into normal C57BL/6NCr mice by i.v. (tail vein) injection of 10^6^ cells/mouse and OT-I cells were identified as CD8^+^Thy1.1^+^ cells. Blood samples were collected at indicated times, and the analysis of memory CTLs was based on samples from spleen and/or blood. Single cell suspensions were prepared, viable cell counts were performed (trypan blue), and the percentage of OT-I cells in the sample was determined by flow cytometry. Background for determining OT-I cell numbers was determined by identical staining of cells from normal C57BL/6 mice (no adoptive transfer). Analysis was done using a FACSCalibur™ flow cytometer and CELLQuest™ software (BD Biosciences) to determine the percentage and total OT-I cells in the samples. Flowjo software (Tree Star Inc.) was used for data analysis.

### Intracellular Cytokine Staining after *in vitro* Stimulation

Spleen cells from adoptively transferred mice were incubated at a concentration of 2 × 10^6^ cells/ml in RP-10 with 0.2 µM OVA_257–264_ peptide and 1 µl Brefeldin A (Biolegend) for 3.5 hrs at 37°C. Cells were fixed in fixing buffer (Biolegend) for 15 min at 4°C, permeablized in Saponin-containing Perm/Wash buffer (Biolegend) for 15 min at 4°C, and stained with PE-conjugated antibody to IFNγ for 30 min at 4°C. Cells were then washed once with Perm/Wash buffer, and once with PBS containing 2% FBS. Staining for Granzyme B (GZB) followed the same procedure as for IFNγ staining except without peptide stimulation.

### Intracellular Staining for Cell Signaling Molecules

Spleen cells from adoptively transferred mice were washed twice with cold PBS (4^o^C), and fixed with 2% paraformaldehyde for 20 min at 37^o^C. The cells were chilled on ice for 2 minutes and washed twice with cold PBS. Permeablization was performed using 90% ice-cold methanol (stored at −20^o^ C) on ice for 30 min. Permeablized cells were washed twice with cold PBS, and blocked for 10 min with 0.5% BSA-PBS at room temperature. Staining with primary and secondary antibodies was carried out for 30 min at 4^o^C. Cells were washed twice with 0.5% BSA-PBS after each staining.

### *In vitro* Stimulation of Naïve OT-I T cells

Naïve OT-I PL T cells were purified as described above and stimulated for a specified time *in vitro* in flat-bottom microtiter wells coated with antigen (DimerX H-2Kb:Ig fusion protein loaded with OVA_257–264_ peptide; BD Pharmingen) and recombinant B7-1/Fc chimeric protein (R&D Systems) as previously described [22], [25]. 3×10^5^ cells in 1.5 ml of Allos media were placed in each well and 2.5 U/ml of IL-2 was added to all wells (24-well plate). Where indicated, cultures were supplemented with 2 U/ml of murine rIL-12 (R&D Systems). Nicotine stock and rapamycin stock were diluted with corresponding culture medium as indicated. Cells that received IL-12 *in vitro* were termed 3SI OT-I, and cells without IL-12 treatment were termed 2SI OT-I. Transferred cells were identified by staining with anti-Thy 1.1 and anti-CD8 mAbs.

### Statistical Analysis

Data was graphed and analyzed using a two-tailed Student's *t* test (GraphPad Prism 5.0 software). Comparisons with a P value of <0.05 were considered significantly different.

## Supporting Information

Figure S1 **Nicotine inhibits CTL memory programming.** A–C: Purified OT1 cells were cultured for 3 days with 3SI in the presence of nicotine at different concentrations. CTLs were harvested and transferred into B6 recipients at 10^6^/mouse. A) Comparison of CD127 expression on memory CTLs in spleen 30 days after transfer. (B–E) Memory CTL heterogeneity in tissues. In vitro stimulated cells with 3SI in the presence or absence of nicotine at 10 µM were transferred into B6 mice for 30 days, and memory OT1 was examined in peripheral lymph nodes, spleen, bone marrow and lung. F. Memory CTL frequency in blood 30 days after transfer (the same as in A).
(DOCX)

Table S1 Mouse and Human nAChR Primers.
(DOCX)
