# Supplementary material for: Nicotine Inhibits Memory CTL Programming
Source: PLoS One. 2013 Jul 2;8(7):e68183. doi: 10.1371/journal.pone.0068183 (PMC3699522; doi:10.1371/journal.pone.0068183)
Supplement: Figure S1 — Nicotine inhibits CTL memory programming. A–C: Purified OT1 cells were cultured for 3 days with 3SI in the presence of nicotine at different concentrations. CTLs were harvested and transferred into B6 recipients at 106/mouse. A) Comparison of CD127 expression on memory CTLs in spleen 30 days after transfer. (B–E) Memory CTL heterogeneity in tissues. In vitro stimulated cells with 3SI in the presence or absence of nicotine at 10 µM were transferred into B6 mice for 30 days, and memory OT1 was examined in peripheral lymph nodes, spleen, bone marrow and lung. F. Memory CTL frequency in blood 30 days after transfer (the same as in A). (DOCX) [file pone.0068183.s001.docx]

**Supplemental Materials**

**
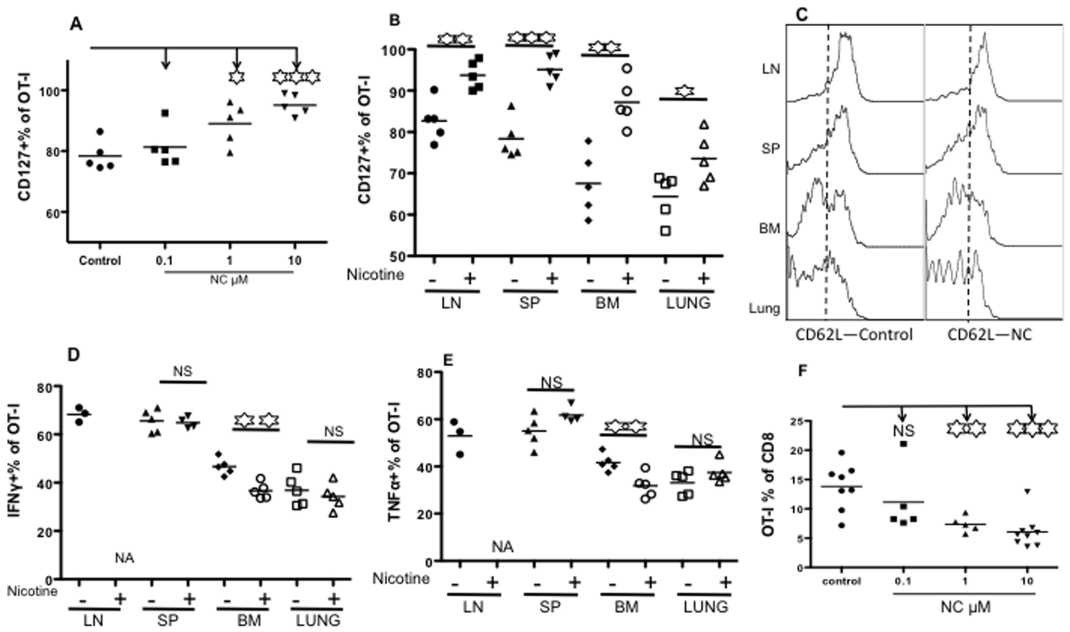
**

**Figure S1. Nicotine inhibits CTL memory programming.** A-C: Purified OT1 cells were cultured for 3 days with 3SI in the presence of nicotine at different concentrations. CTLs were harvested and transferred into B6 recipients at 10^6^/mouse. A) Comparison of CD127 expression on memory CTLs in spleen 30 days after transfer. (B-E) Memory CTL heterogeneity in tissues. In vitro stimulated cells with 3SI in the presence or absence of nicotine at 10 μM were transferred into B6 mice for 30 days, and memory OT1 was examined in peripheral lymph nodes, spleen, bone marrow and lung. F. Memory CTL frequency in blood 30 days after transfer (the same as in A).
