# Supplementary material for: Nicotine Inhibits Memory CTL Programming
Source: PLoS One. 2013 Jul 2;8(7):e68183. doi: 10.1371/journal.pone.0068183 (PMC3699522; doi:10.1371/journal.pone.0068183)
Supplement: Table S1 — Mouse and Human nAChR Primers. (DOCX) [file pone.0068183.s002.docx]

**Table S1**. **Mouse and Human nAChR Primers**

| Species | Name of Primer | Sequences |
| --- | --- | --- |
| Mouse | Mouse α1 nAChR-F | gtatgagaacgcgcacagaa |
|  | Mouse α1 nAChR-R | acaaaacaaaaaggcgatgg |
|  | Mouse α2 nAChR-F | aagctcagccccacttatca |
|  | Mouse α2 nAChR-R | acacccatggaagagtctgg |
|  | Mouse α3 nAChR-F | ccgctgttctacaccatcaa |
|  | Mouse α3 nAChR-R | gtcaccttctccccacagtc |
|  | Mouse α4 nAChR-F | ccaactggacttctgggaaa |
|  | Mouse α4 nAChR-R | aaggcgtaggtgatgtcagg |
|  | Mouse α5 nAChR-F | ctccatgaaattcggctcat |
|  | Mouse α5 nAChR-R | cgctcatgatttcccattct |
|  | Mouse α6 nAChR-F | gctgctggcttgatcttctc |
|  | Mouse α6 nAChR-R | aaggaccccaaaagccatac |
|  | Mouse α9 nAChR-F | ctctgccttcaagccaaatc |
|  | Mouse α9 nAChR-R | tcccctctggtaagctgaga |
|  | Mouse α10 nAChR-F | cttgcctctcccctaattcc |
|  | Mouse α10 nAChR-R | tcctgtctcagcctccttgt |
|  | Mouse β1 nAChR-F | agcctgaacgagaaggatga |
|  | Mouse β1 nAChR-R | agcagtgatgcggagagaat |
|  | Mouse β2 nAChR-F | attccaatgctgtggtctcc |
|  | Mouse β2 nAChR-R | gtgcagttctgctggtcaaa |
|  | Mouse β3 nAChR-F | taaaggttccatcggagtcg |
|  | Mouse β3 nAChR-R | gttccgctggatttcacaat |
|  | Mouse β4 nAChR-F | tgctggcactcacattcttc |
|  | Mouse β4 nAChR-R | acacagtggtgacgatggaa |
|  | Mouse γ nAChR-F | cctcctgctccatctctgtc |
|  | Mouse γ nAChR-R | cgggtcaatgaagatccact |
|  | Mouse δ nAChR-F | tcacagagaacggtgagtgg |
|  | Mouse δ nAChR-R | cggcggatgataaggtagaa |
|  | Mouse ε nAChR-F | cattgacacggcagctttta |
|  | Mouse ε nAChR-R | tccaggaccttctgtggaac |
|  | Mouse GAPDH-F | tgtctcctgcgacttcaacagc |
|  | Mouse GAPDH-R | tgtaggccatgaggtccaccac |
| Human | Human α2 nAChR-F | cagatggagcagactgtgga |
|  | Human α2 nAChR-R | gtaggtgacgtcggggtaga |
|  | Human α3 nAChR-F | accatgaagttcggttcctg |
|  | Human α3 nAChR-R | tatagcctggggctttgatg |
|  | Human α4 nAChR-F | catcacctatgccttcgtca |
|  | Human α4 nAChR-R | ggcaggtagaagaccagcac |
|  | Human α5 nAChR-F | tctggacaccagacatcgtt |
|  | Human α5 nAChR-R | ggtggagtccaggtgacagt |
|  | Human α7 nAChR-F | atcgatgtacgctggtttcc |
|  | Human α7 nAChR-R | ccactaggtcccattctcca |
|  | Human α9 nAChR-F | ctgggagtgaccatcctgtt |
|  | Human α9 nAChR-R | ggccatcgtggctatgtagt |
|  | Human α10 nAChR-F | tccaggccacctgagttatc |
|  | Human α10 nAChR-R | ggaaggtattggcaatggtg |
|  | Human β1 nAChR-F | caaacccgagagagacctga |
|  | Human β1 nAChR-R | acctattgggtttggggaag |
|  | Human β2 nAChR-F | ctattccaatgccgtggtct |
|  | Human β2 nAChR-R | gtccacgaacggaacttcat |
|  | Human γ nAChR-F | gctaaccctcaccaacctca |
|  | Human γ nAChR-R | ccacaggccttcgtagtctc |
|  | Human δ nAChR-F | aacggggagtgggagatagt |
|  | Human δ nAChR-R | gcaccaggatgttgatgatg |
|  | Human ε nAChR-F | cctgacccttccacagtcat |
|  | Human ε nAChR-R | taggcagagagagcctccag |
|  | Human GAPDH-F | cggctactagcggttttacg |
|  | Human GAPDH-R | gctgcgggctcaatttatag |
